# The versatile roles of odontogenic ameloblast-associated protein in odontogenesis, junctional epithelium regeneration and periodontal disease

**DOI:** 10.3389/fphys.2022.1003931

**Published:** 2022-09-02

**Authors:** Sipin Zhu, Chuan Xiang, Oscar Charlesworth, Samuel Bennett, Sijuan Zhang, Maio Zhou, Omar Kujan, Jiake Xu

**Affiliations:** ^1^ Department of Orthopaedics, The Second Affiliated Hospital and Yuying Children’s Hospital of Wenzhou Medical University, Wenzhou, China; ^2^ Molecular Lab, School of Biomedical Sciences, University of Western Australia, Perth, Western Australia, Australia; ^3^ Department of Orthopaedics, The Second Hospital of Shanxi Medical University, Taiyuan, China; ^4^ Affiliated Stomatology Hospital of Guangzhou Medical University, Guangdong Engineering Research Center of Oral Restoration and Reconstruction, Guangzhou Key Laboratory of Basic and Applied Research of Oral Regenerative Medicine, Guangzhou, China; ^5^ Department of Stomatology, Guangdong Provincial People’s Hospital, Guangdong Academy of Medical Science, Guangzhou, China; ^6^ UWA Dental School, The University of Western Australia, Perth, Western Australia, Australia

**Keywords:** epithelium regeneration, dental disorders, odontogenic ameloblast-associated, junctional epithelium, enamel-related

## Abstract

Junctional epithelium (JE) is a vital epithelial component which forms an attachment to the tooth surface at the gingival sulcus by the adhesion of protein complexes from its basal layer. Disruption of the JE is associated with the development of gingivitis, periodontal disease, and alveolar bone loss. Odontogenic ameloblast-associated (ODAM) is comprised of a signal peptide and an ODAM protein with 12 putative glycosylation sites. It is expressed during odontogenesis by maturation stage ameloblasts and is incorporated into the enamel matrix during the formation of outer and surface layer enamel. ODAM, as a secreted protein which is accumulated at the interface between basal lamina and enamel, mediates the adhesion of the JE to the tooth surface; and is involved with extracellular signalling of WNT and ARHGEF5-RhoA, as well as intracellular signalling of BMP-2-BMPR-IB-ODAM. ODAM is also found to be highly expressed in salivary glands and appears to have implications for the regulation of formation, repair, and regeneration of the JE. Bioinformatics and research data have identified the anti-cancer properties of ODAM, indicating its potential both as a prognostic biomarker and therapeutic target. Understanding the biology of ODAM will help to design therapeutic strategies for periodontal and dental disorders.

## Introduction

Junctional epithelium (JE) essentially consists of stratified squamous nonkeratinized epithelium and forms an epithelial component of the dentogingival junction, which attaches the gingiva to the tooth surface and is involved in defence against gram-negative anaerobic bacteria infection. During the progression of gingivitis to periodontitis, the JE becomes progressively compromised over time, migrates apically and forms pocket epithelium, leading to impaired physical barriers against periodontal infection. The JE represents an important component for periodontal tissue regeneration. Formation of the JE from cells of the reduced enamel epithelium begins during tooth eruption to the oral cavity, where the JE fuses with the oral epithelium to form the primary JE, which is subsequently replaced by a JE derived from the gingival epithelium (Nanci and Bosshardt 2006). The JE contains ongoing differentiating epithelial cells which do not keratinise, and recent research contends that the JE originates from the odontogenic epithelium and is not replaced by oral epithelium during tooth eruption, but rather more gradually after the tooth has erupted (Bosshardt and Lang 2005; Yajima-Himuro et al., 2014).

Odontogenic ameloblast-associated protein (ODAM), also referred to as APIN, is expressed in the JE as an enamel-related gene (Hammarstrom 1997; Park et al., 2007; Fan et al., 2015; Nakayama et al., 2015), which also includes amelotin (AMTN) (Iwasaki et al., 2005; Moffatt et al., 2006), and secretory calcium-binding phosphoprotein proline-glutamine rich (SCPPPQ1) from the secretory calcium-binding phosphoprotein (SCPP) cluster (Kawasaki 2009; Moffatt et al., 2014; Fouillen et al., 2017). ODAM protein is highly conserved in mammals (Moffatt et al., 2008). Its discrete localisation in the atypical basal lamina (BL) connecting epithelial cells to tooth surfaces further suggests that ODAM plays a role in the regulation of adhesive processes in the interface of epithelial cells to the tooth (Moffatt et al., 2008; Nishio et al., 2010a; Dos Santos Neves et al., 2012).

Under pathological conditions of periodontitis and peri-implantitis, ODAM is considered a potential biomarker of disease. The research found that following the loss of JE attachment caused by diseases, ODAM expression was absent in JE, and ODAM was detected in gingival crevicular fluid (Lee, et al., 2015a; Lee et al., 2018). As the periodontitis progresses to periodontal disease, it becomes incurable. To date, the role of ODAM in the pathological process of periodontal diseases remains to be fully elucidated.

Here we review the molecular structure, expression, and role of ODAM in odontogenesis, regeneration of JE, and periodontal diseases. We also discuss putative binding partners of ODAM as an intracellular molecule or as an extracellular secreted factor. Understanding the role of ODAM will facilitate the regeneration of impaired JE, which is highly important to the treatment of periodontal diseases.

## Molecular structure and expression of odontogenic ameloblast-associated protein

**FIGURE 1:**
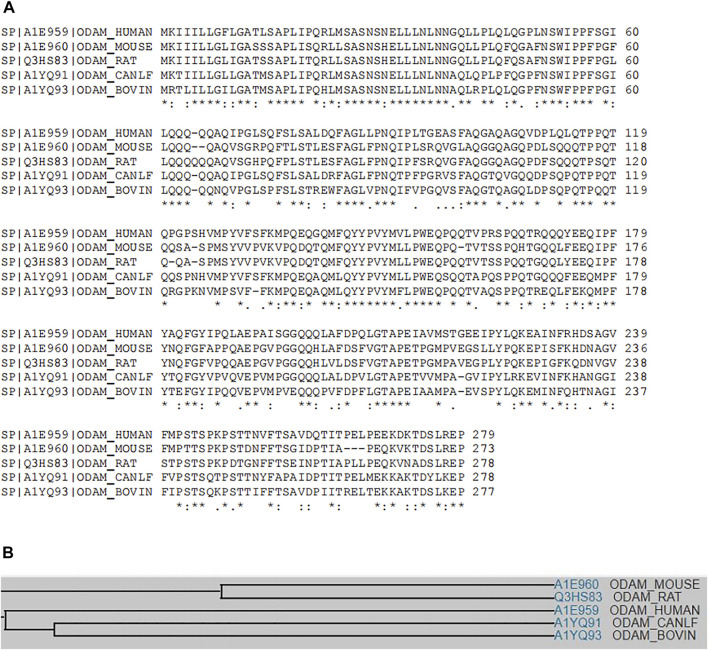
**(A)** Multiple sequence alignment analyses show that ODAM shares sequence similarity and identity among various species including mouse, human, rat, dog, and bovine (https://www.uniprot.org/align). **(B)** A family tree of ODAM protein homologues is elucidated.

Multiple sequence alignment analysis results show that human ODAM has substantial sequence identity or similarity to mouse, rat, dog and bovine ODAM (Figures 1A,B), indicating conserved nature among various species (Moffatt et al., 2008). However, ODAM has lack of sequence homology with other odontogenesis-related proteins including AMTN, amelogenin X-linked (AMELX), ameloblastin (AMBN), and enamelin (ENAM) (data not shown), suggesting its unique function in odontogenesis.

**FIGURE 2:**
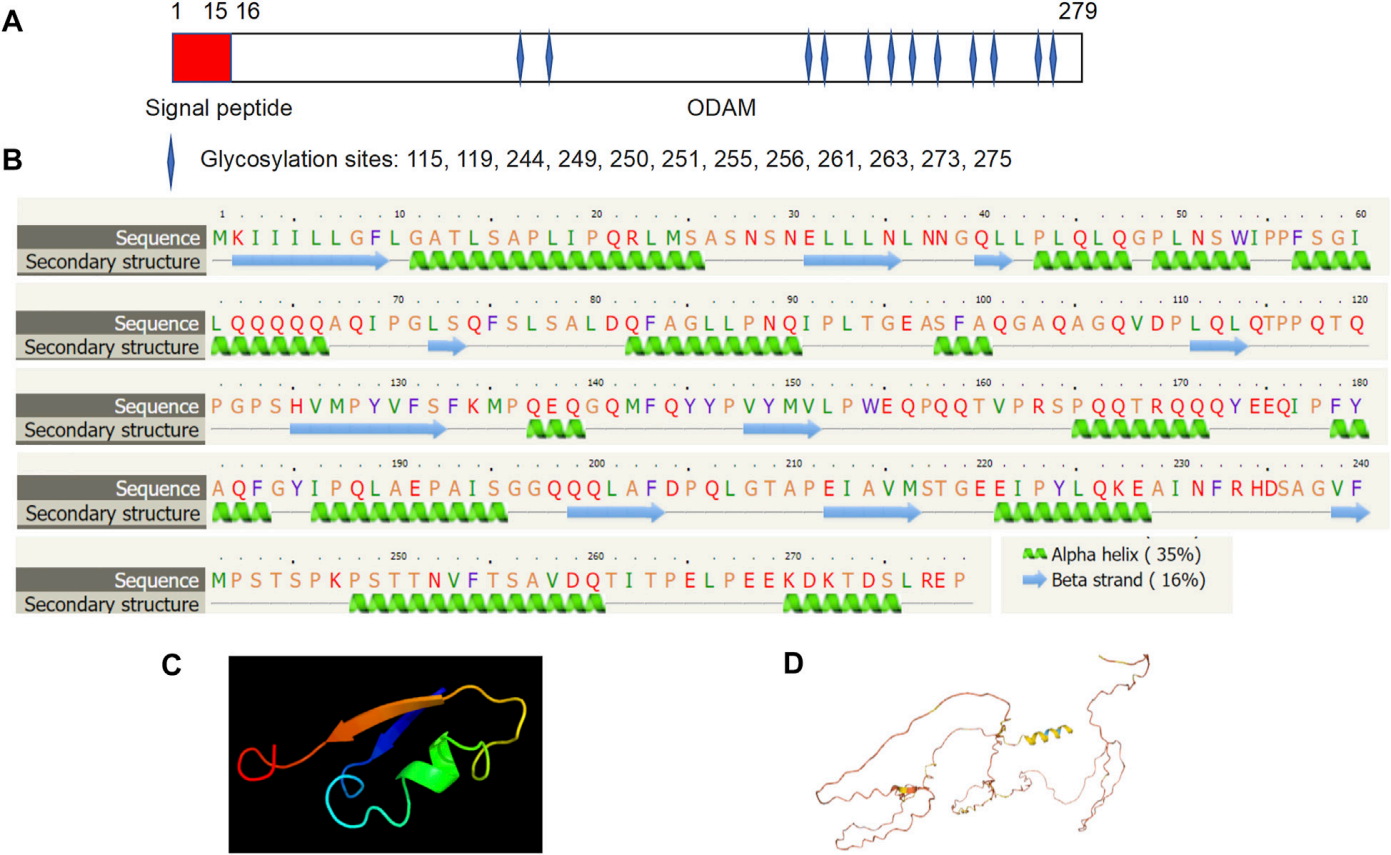
Molecular structure of ODAM, predicted by bioinformatics information based on Uniprot (https://www.uniprot.org/), showing that ODAM is comprised of a signal peptide and an ODAM protein with 12 putative glycosylation sites at amino acid residues 115, 119, 244, 249, 250, 251, 255, 256, 261, 263, 273, and 275, respectively **(A)**. Secondary structure prediction showed that ODAM has characteristics of 10 beta-sheet domains and 14 alpha-helix based on bioinformatic analysis **(B)**. Predicted 3D structures of ODAM are shown by using web-based Phyre2 **(C)**, and AlphaFold web portals **(D)**.

Molecular structure analysis revealed that human ODAM is comprised of a signal peptide (amino acid residues 1–15) and an ODAM protein (amino acid residues 16–279) with 12 putative glycosylation sites at amino acid residues 115, 119, 244, 249, 250, 251, 255, 256, 261, 263, 273, and 275 respectively (Figure 2A). Secondary structure prediction showed that ODAM has characteristics of 10 beta-sheet domains and 14 alpha-helix based on bioinformatic analyses (Figure 2B). Further, 3D structures of ODAM are also shown using the Phyre2 (Kelley et al., 2015) (Figure 2C), and AlphaFold web-based portals (Jumper et al., 2021) (Figure 2D).

Gene expression studies showed that ODAM consistently is found to be expressed within the JE (Redd and Byers 1994; Nishio et al., 2010b), in cell clusters located between oral epithelium and the erupting tooth (Nishio et al., 2010a). During JE regeneration, ODAM was found to be expressed at the leading wound edge initially, and then in the regenerating JE and isolated cell clusters of the subjacent connective tissue at the tooth interface (Nishio et al., 2010b), which suggests ODAM is involved in the cellular signalling events that occur during formation and regeneration of the JE. Consistently, immunolabeling showed that ODAM is localized to the atypical BL of enamel (Dos Santos Neves et al., 2012). In addition, ODAM was also detected in human salivary gland and trachea (Kestler et al., 2008).

**FIGURE 3:**
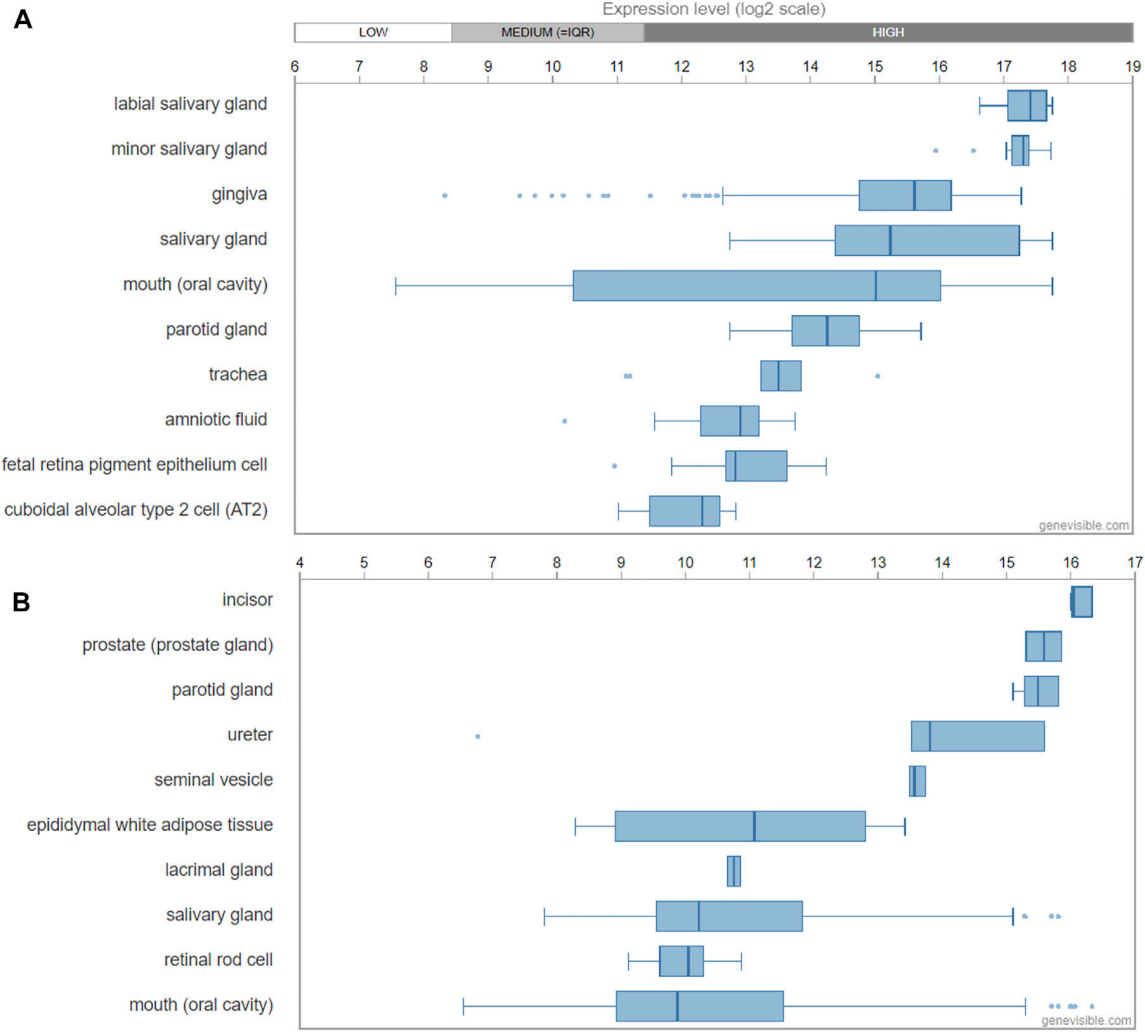
Transcript expression of ODAM analysed by Genevisible® (www.genevisible.com), showing 10 tissues highly expressing human ODAM gene **(A)** and mouse ODAM gene **(B)**.

Genevisible®- based bioinformatics analyses revealed that ODAM mRNA expression was high in the labial salivary gland, minor salivary gland, gingiva, salivary gland, mouth (oral cavity), and parotid glands in human tissues (Figure 3A); While in mice, ODAM is highly expressed by incisor, prostate (prostate gland), parotid gland, ureter, seminal vesicle, epididymal white adipose tissue, lacrimal gland, and salivary gland tissues (Figure 3B) (Hruz et al., 2008). Its consistently high expression in both human and mouse salivary glands is an interesting observation, which requires further investigation of its functional significance, and prompts the question to future research on the various roles and pleiotropy of ODAM in a tissue-specific manner (Fagerberg et al., 2014). For instance, valuable regulatory insights linking ODAM expression during salivary gland formation to the regulation of odontogenesis and JE structure could have critical potential applications for regenerative medicine. Surprisingly, ODAM was also highly expressed in prostate glands, which suggests that ODAM might be involved with the repair of prostate epithelial cells damaged by local infection and environmental factors, thereby playing a similar protective role as in the JE. Further experiments will be required to address these intriguing lines of enquiry.

## Putative odontogenic ameloblast-associated protein receptor and binding partners

ODAM interacts with various molecules as an intracellular protein and binds to an unknown receptor as a predicted secreted factor. In ameloblasts, intracellular ODAM was found to interact with bone morphogenetic protein (BMP) receptor type-IB (BMPR-IB) via the C-terminus of ODAM, resulting in increased ODAM phosphorylation induced by BMP-2 via MAPKs (Lee, et al., 2012a). This suggests that intracellular BMP-2-BMPR-IB-ODAM signalling has important role in ameloblast differentiation and enamel mineralization (Lee, et al., 2012b). Further, the interaction of BMPR-IB with ODAM appears to be independent of BMP-2, indicating that BMPR-IB is a mediator of the BMP-2-induced phosphorylation of ODAM (Lee, et al., 2012a). In addition, ODAM was found to affect the attachment of the JE to the tooth surface through its interaction with Rho guanine nucleotide exchange factors 5 (ARHGEF5) and the expression of downstream factors such as Rho-associated coiled-coil containing protein kinase (ROCK), and the induction of Ras Homolog Family Member A (RhoA) activity (Lee, et al., 2015a). ODAM-ARHGEF5-RhoA signalling is potentially mediated by fibronectin/laminin-integrin, and impairment of this signalling in JE was associated with periodontal diseases, which is suggestive of a role of ODAM as an intracellular protein (Lee, et al., 2015b). As a secreted factor, the receptor of ODAM is unknown and remains to be identified; whilst the mechanisms by which ODAM promotes the activation of a ARHGEF5-RhoA signalling pathway remains to be elucidated.

**FIGURE 4:**
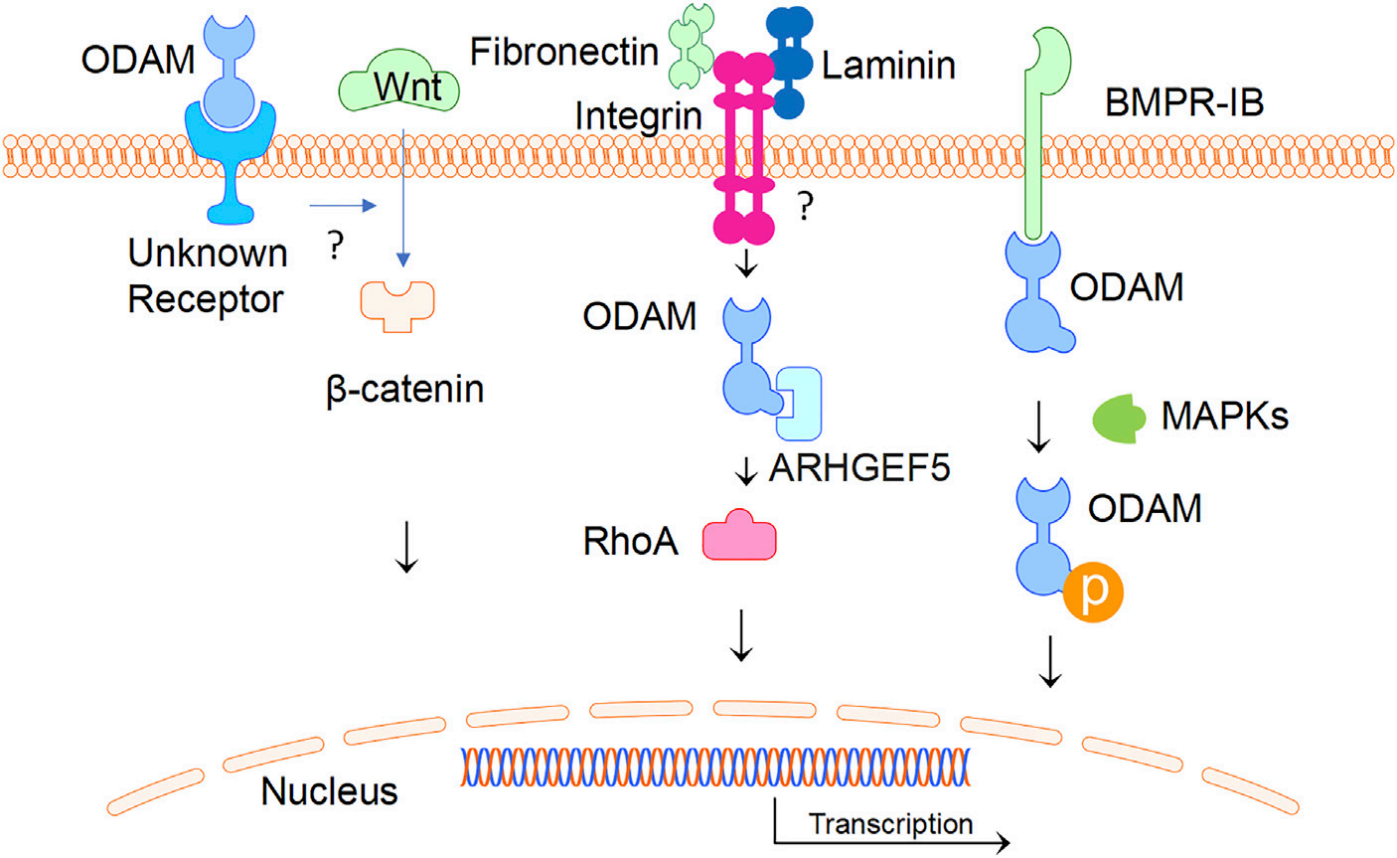
ODAM and its interactive partners. ODAM binds and activates intercellular signalling molecules, leading to various cellular activities. In addition, ODAM binds cell surface unknown receptor and mediates JE homeostasis.

Interestingly, ODAM was detected extracellularly in the atypical BL, which formulates the attachment of matured ameloblasts and JE cells to the tooth (Moffatt et al., 2008; Dos Santos Neves et al., 2012; Nishio et al., 2013). As a predicted matricellular protein, ODAM might have both cellular and matrix properties (Wazen et al., 2015). Most recently, ODAM was found to activate the WNT signalling pathway as measured by a luciferase reporter gene assay (Song et al., 2021). Nevertheless, a question remains as to how ODAM mediates intracellular signalling as a predicted secreted protein. Further investigations will be required to identify ODAM receptor leading to the activation of WNT or receptor signalling pathways (Figure 4).

## The role of odontogenic ameloblast-associated protein in odontogenesis

ODAM is one of five ameloblast-secreted proteins (Sire et al., 2007), and is suggested to serve as a tooth-associated epithelial protein to regulate odontogenesis for the initiation and generation of the tooth. ODAM was also found to be expressed in the enamel organ (Kestler et al., 2008) and appears to be incorporated in the enamel matrix during the maturation phase for the formation of outer and surface layer enamel (Somogyi-Ganss et al., 2012). Further research is suggestive of ODAM expression in rodent enamel organ for the regulation of dental development by maturation stage ameloblasts (Moffatt et al., 2008; Dos Santos Neves et al., 2012). In addition, ODAM was found to be predominantly expressed and secreted extracellularly by ameloblasts in association with MMP-20 for the modulation of enamel mineralization (Lee et al., 2010; Lee, et al., 2012a); and ODAM was shown to induce hydroxyapatite mineralization by a dose-dependent manner in simulated body fluid (Ikeda et al., 2018). In line with this, Amel- and Ambn-deficient calvariae and calvarial osteoblast cultures showed a reduction in mineralized nodule accumulation, together with a reduction in the expression of Runx2, Sp7, Ibsp, and Msx2, which is indicative of their possible roles in bone mineralization (Atsawasuwan et al., 2013). However, research found that no substantial alterations in enamel layer surface were observed in ODAM deficient mice (Wazen et al., 2015), and that no major effect on enamel was observed in Amtn KO mice, which together indicates ODAM plays a subtle role in amelogenesis of surface enamel (Nakayama et al., 2015; Wazen et al., 2015). Further research is required to clarify the vital function of ODAM for amelogenesis and to investigate its potential role in skeletal homeostasis.

Interestingly, a patient with a loss-of-function mutation of SPARC related modular calcium binding 2 (SMOC2) - a secreted matricellular protein - was found to have dental and skeletal abnormalities, including oligodontia, microdontia, tooth root anomalies, alveolar bone hypoplasia, and skeletal deformities (Morkmued et al., 2020). Whilst Smoc2 (−/−) mice showed dental anomalies of reduced tooth number, decreased size, malformed enamel, and altered RNA sequencing data with dysregulation of enamel and dentine matrix genes, including ODAM (Morkmued et al., 2020). Additional findings suggested SMOC2 would inhibit injury-induced jawbone osteonecrosis and periodontal decay (Morkmued et al., 2020). Further research is necessary to illuminate the mechanistic pathways connecting ODAM and SMOC2 for their apparent function in dental and skeletal homeostasis and disease.

The potential role of single nucleotide variants of ODAM, MMP20, and AMELX in the development of dental fluorosis was recently investigated using DNA sequencing. Results from this study indicated rs1514392 of ODAM, did not have a significant effect on the severity of dental fluorosis between populations, whilst rs1784418 of MMP20 was significantly associated with susceptibility to dental fluorosis (Tremillo-Maldonado et al., 2020). Given the apparent functional correlation of ODAM and MMP20 (Lee et al., 2010; Lee, et al., 2012b), future research from a larger and broader population sample could provide additional findings of genetic susceptibility to dental fluorosis which might explain the observed phenotypic variation.

## The role of odontogenic ameloblast-associated protein in the integrity of junctional epithelium

JE is interposed between the depth of the gingival sulcus and mineralized tissues of the tooth surface, where it is attached by the epithelial attachment. The JE is covered by biofilm and the deepest layer undergoes continuous and rapid cell division to aid in the protective function of the JE against periodontal disease. The expression of ODAM in JE has stimulated further interest of its role in maintaining the integrity of the JE. ODAM was shown to be expressed throughout the JE and is thought to play an important role at the cell-tooth interface, both during formation and regeneration of the JE (Nishio et al., 2010a). ODAM was found to be expressed by activated epithelial cell rests of Malassez (ERM) at early time points of three and 5 days following iatrogenic periodontal challenge in a rodent model, indicating that ODAM appears to be a vital component of the molecular and cellular signalling events which occur for repair and regeneration of the JE and periodontium (Nishio et al., 2010b). Additional findings from an Odam knockout (KO) mouse model replicated structural changes observed during human periodontal disease, suggesting that ODAM is involved in the maintenance of periodontal structural integrity (Wazen et al., 2015). For instance, Odam KO mice showed slower healing of the JE following gingivectomy than wild type mice, and the JE was found more easily detached from teeth, indicative of a role of ODAM in JE regeneration and attachment (Wazen et al., 2015). As distinct from AMTN and SCPPPQ1, two functionally related proteins of the SCPP cluster which are also present in the BL, ODAM is expressed continuously and early throughout the JE (Nishio et al., 2010a; Moffatt et al., 2014). Unlike the Amtn KO mouse in which the integrity of the JE was largely unaffected (Nakayama et al., 2015), Odam KO was shown to have an obvious effect on the JE and periodontal condition of aged mice (Wazen et al., 2015). The discrepancy between Odam and Amtn KO phenotypes suggest that ODAM and AMTN are expressed differentially. ODAM is expressed at cell-tooth interfaces and among the cells of the JE (Wazen et al., 2015), whereas AMTN is expressed at the cell-tooth interface (Nishio et al., 2010b; Sawada et al., 2014) and during regeneration of the JE (Nishio et al., 2010a). Collectively, ODAM appears to be involved with the regulation of adhering of the epithelial cells of the JE to the tooth surface, activation of ERM, and repair and regeneration of the JE, thereby maintaining the integrity of the tooth-JE interface and protecting periodontal tissues from further damage by the extraneous oral environment. Future research could develop therapeutic applications targeting ODAM for periodontal tissue regeneration of the JE.

## Possible role of odontogenic ameloblast-associated protein in tumours

ODAM is postulated to be an inhibitor of cancer onset and progression, whilst its potential as a therapeutic target and biomarker remains incompletely characterized. Initially, ODAM was found in calcifying epithelial odontogenic tumour-associated amyloid (Solomon et al., 2003). ODAM was subsequently characterized as the main protein constituent of a unique form of odontogenic amyloid found in calcifying epithelial odontogenic, or Pindborg, tumours (Murphy et al., 2008). ODAM was also found to be overexpressed in several cancers of epithelial origin, such as gastric, breast, salivary gland, trachea, and colorectal cancers indicating its potential both as a biomarker for prognosis and therapeutic target (Aung et al., 2006; Kestler et al., 2008; Siddiqui et al., 2009; Kestler et al., 2011; Yu et al., 2016). For example, significant serum titres of anti-ODAM IgG antibodies were detected patients with epithelial neoplasms from gastric, lung, and breast origin (Kestler et al., 2008). ODAM was also found to attenuate breast cancer cell metastasis by regulating RhoA signalling and the rearrangement of actin filaments (Lee, et al., 2015b). Whilst further research indicated ODAM expression could be a predictive marker of breast cancer survival (Siddiqui et al., 2009). Moreover, upregulated ODAM expression was found to inhibit the neoplastic properties of breast cancer cells, suggesting its potential clinical importance for tumour therapy (Kestler et al., 2011). Additional research of the anti-neoplastic effects of ODAM was shown in melanoma and breast cancer cell cultures, where increased ODAM expression was accompanied by increased expression of the tumour suppressor gene, phosphatase and tensin homolog on chromosome 10 (PTEN), and decreased AKT expression (Foster et al., 2013). In gastrointestinal stromal tumours, the risk of recurrence was found to be correlated with cytoplasmic, and not nuclear, expression of ODAM and phosphorylated (activated) AKT (McLoughlin et al., 2016). ODAM expression level was found to be suggestive of the onset and patient prognosis of colorectal carcinoma (CRC), which was also associated with upregulation of PTEN and inactivation of AKT signalling (Yu et al., 2016). It is postulated that the pathogenic role of ODAM in tumours might be related to its intracellular, rather than extracellular secreted form.

## Summary

**FIGURE 5:**
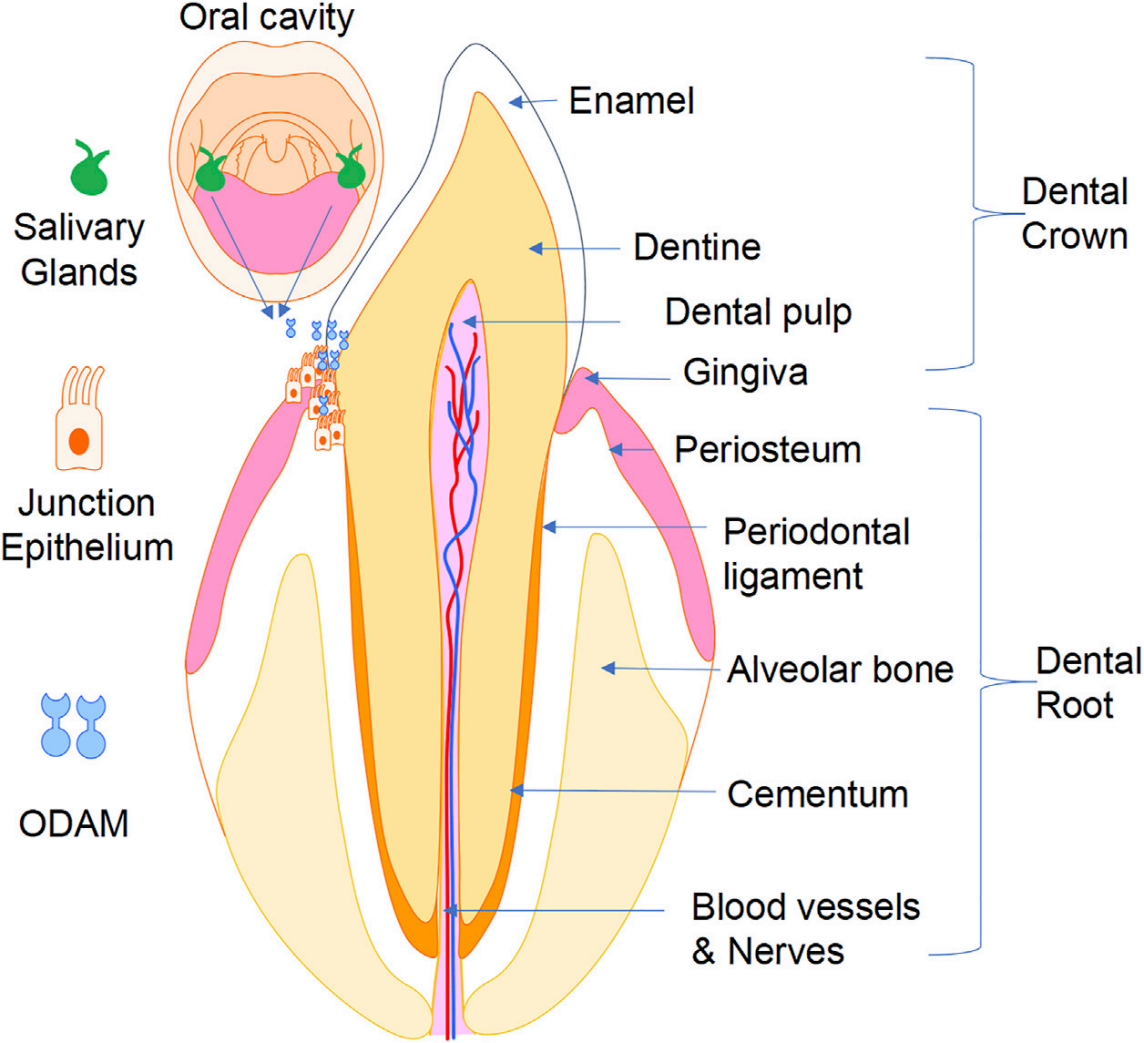
A working model showing that ODAM plays a role in odontogenesis and maintaining integrity of the JE, as well as its potential pathological role in the development of gingivitis, periodontitis, and erosion of underlying dentoalveolar bone.

Proper adhesion of the JE to the tooth surface is crucial for maintaining periodontal health by sealing the periodontal tissues and preventing their infection by bacteria from the oral cavity (Figure 5). Impairment of JE attachment contributes to the development of gingivitis, periodontitis, and dentoalveolar bone loss. ODAM was found to be predominantly expressed by ameloblasts and JE, and to play various roles in odontogenesis, formation, repair, and regeneration of the JE, and in the level of local inflammatory response of the periodontal tissues. While the precise tissue-specific roles of ODAM remain to be completely elucidated, its role in maintaining the integrity of the JE, together with its effects on dedifferentiating epithelial neoplastic cells, suggest that ODAM could be an important modulator of cell status, a diagnostic biomarker, and therapeutic target. Further studies will be needed to uncover the molecular and cellular signalling mechanisms of ODAM in the pathobiology of these disorders, and to facilitate the development of ODAM as a novel target for therapeutic applications.
